# Correction: The sarcoma ring trial: a case-based analysis of inter-center agreement across 21 German-speaking sarcoma centers

**DOI:** 10.1007/s00432-025-06193-y

**Published:** 2025-04-24

**Authors:** Siyer Roohani, Jolina Handtke, Kamal Hummedah, Markus Albertsmeier, Dimosthenis Andreou, Leonidas Apostolidis, Marinela Augustin, Sebastian Bauer, Moritz Billner, Florian Bösch, Christoph K. W. Deinzer, Niklas Deventer, Anna Duprée, Franziska Eckert, Lars Engel, Katja Fechner, Hagen Fritzsche, Verena Gaidzik, Saeed Ghani, Robert Grützmann, Wiebke K. Guder, Rainer Hamacher, Judith S. Hecker, Anne Hendricks, Axel Hillmann, Philipp Houben, Georg Hübner, Philipp Ivanyi, Christina Jentsch, Maren Jordan, Peter Kappl, Moritz Kaths, Torsten Kessler, Johanna Kirchberg, Carolin Knebel, Robert Krempien, Burkhard Lehner, Ulrich Lenze, Lars H. Lindner, Alisa Martina Lörsch, Nadia Maguire, Sophie Müller, Pompiliu Piso, Vlatko Potkrajcic, Peter Reichardt, Stephan Richter, Simone Schewe, Lars M. Schiffmann, Felicitas Scholten, Jana Käthe Striefler, Matthias Schwarzbach, Katharina Seidensaal, Sabine Semrau, Joanna Szkandera, Christoph J. Szuszies, Beate Timmermann, Armin Tuchscherer, Armin Wiegering, Moritz T. Winkelmann, David Kaul, Jens Jakob

**Affiliations:** 1 Department of Radiation Oncology, Charité-Universitätsmedizin Berlin, Corporate Member of Freie Universität Berlin and Humboldt-Universität zu Berlin, Berlin, Germany; 2 Berlin Institute of Health at Charité-Universitätsmedizin Berlin, BIH Biomedical Innovation Academy, BIH Charité (Junior) Clinician Scientist Program, Berlin, Germany; 3 German Cancer Consortium (DKTK), Partner Site Berlin, a Partnership Between DKFZ and Charité-Universitätsmedizin Berlin, Berlin, Germany; 4 Sarcoma Unit, Department of Surgery, University Medical Center and Medical Faculty Mannheim, University of Heidelberg, Mannheim, Germany; 5 DKFZ Hector Cancer Institute at the University Medical Center Mannheim, Mannheim, Germany; 6 Department of General, Visceral and Transplantation Surgery, Ludwig-Maximilians-Universität (LMU) Munich, LMU University Hospital, Munich, Germany; 7 Department of Orthopedics and Trauma, Medical University of Graz, Graz, Austria; 8 Department of Orthopedic Oncology, West German Cancer Center, University Hospital Essen, University Duisburg-Essen, Essen, Germany; 9 Department of Medical Oncology, Heidelberg University Hospital, National Center for Tumor Diseases (NCT) Heidelberg, Heidelberg, Germany; 10 Department of Hematology and Oncology, Paracelsus Medical University, Nuremberg, Germany; 11 Department of Medical Oncology and Sarcoma Center, West German Cancer Center, University Duisburg-Essen, Medical School, Essen, Germany; 12 DKTK Partner Site Essen, German Cancer Consortium (DKTK), Heidelberg, Germany; 13 Department of Plastic, Reconstructive and Hand Surgery, Center for Severe Burn Injuries, Paracelsus Medical University, Klinikum Nürnberg, Nuremberg, Germany; 14 Department of General, Visceral and Pediatric Surgery, University Medical Center Göttingen, Göttingen, Germany; 15 Department of Internal Medicine VIII-Medical Oncology and Pneumology, University Hospital Tübingen, Tübingen, Germany; 16 Department for General Orthopaedics and Tumor Orthopaedics, University Hospital Muenster, Muenster, Germany; 17 Department of General, Visceral and Thoracic Surgery, University Medical Center Hamburg-Eppendorf, Hamburg, Germany; 18 Department of Radiation Oncology, University Hospital Tübingen, Tübingen, Germany; 19 Department of Radiation Oncology, AKH, Comprehensive Cancer Center Vienna, Medical University Vienna, Vienna, Austria; 20 Department of Visceral-Thoracic and General Surgery, Paracelsus Medical University, Nuremberg, Germany; 21 Department of Surgery, University Hospital, Erlangen, Germany; 22 University Center for Orthopedics, Trauma and Plastic Surgery, Faculty of Medicine and University Hospital Carl Gustav Carus, TUD, Dresden University of Technology, Dresden, Germany; 23 Clinic for Internal Medicine III, University Hospital Ulm, Ulm, Germany; 24 Helios Klinikum Berlin Buch, Sarkomzentrum, Berlin, Germany; 25 Department of General and Visceral Surgery, Friedrich-Alexander-University, Erlangen, Germany; 26 Department of Medicine III, School of Medicine and Health, Technical University of Munich (TUM), Munich, Germany; 27 Department of General, Visceral, Transplantation, Vascular, and Pediatric Surgery and Comprehensive Cancer Center Mainfranken Würzburg, University Hospital Würzburg, Würzburg, Germany; 28 Department of Orthopedic and Trauma Surgery, Barmherzige Brüder Regensburg Medical Center, Regensburg, Germany; 29 Department for General-, Visceral- and Transplant Surgery, University Hospital Muenster, Muenster, Germany; 30 Department of Radiation Oncology, Barmherzige Brüder Regensburg Medical Center, Regensburg, Germany; 31 Clinic for Hematology, Hemostaseology, Oncology and Stem Cell Transplantation, Hannover Medical School, Hannover, Germany; 32 Department of Radiotherapy and Radiation Oncology, Faculty of Medicine and University Hospital Carl Gustav Carus, TUD, Dresden University of Technology, Dresden, Germany; 33 OncoRay-National Center for Radiation Research in Oncology, Dresden, Germany; 34 Nationales Centrum Für Tumorerkrankungen Dresden (NCT/UCC): Deutsches Krebsforschungszentrum (DKFZ), Universitätsklinikum Carl Gustav Carus Dresden, Medizinische Fakultät der Technischen Universität Dresden, Helmholtz-Zentrum Dresden-Rossendorf (HZDR), Dresden, Germany; 35 Varisano Klinikum Frankfurt Höchst, Frankfurt, Germany; 36 Department of Radiology, Neuroradiology, and Nuclear Medicine, Barmherzige Brüder Regensburg Medical Center, Regensburg, Germany; 37 Department of General, Visceral and Transplantation Surgery, Sarcoma Center, West German Cancer Center, University Hospital Essen, University Duisburg-Essen, Essen, Germany; 38 Department of Hematology and Oncology, University Hospital Muenster, Muenster, Germany; 39 Department of General, Visceral, Thoracic and Vascular Surgery, Faculty of Medicine and University Hospital Carl Gustav Carus, TUD Dresden University of Technology, Dresden, Germany; 40 Department of Orthopaedics and Sports Orthopaedic, Klinikum Rechts Der Isar, Technical University of Munich (TUM), Munich, Germany; 41 Clinic for Radiotherapy, HELIOS Klinikum Berlin-Buch, Schwanebecker, Berlin, Germany; 42 MSB Medical School Berlin, Fakultät für Medizin, Berlin, Germany; 43 Department of Orthopaedics, Heidelberg University Hospital, Heidelberg, Germany; 44 SarKUM, Center of Bone and Soft Tissue Tumors, LMU University Hospital, LMU Munich, 81377 Munich, Germany; 45 Department of Medicine III, LMU University Hospital, LMU Munich, 81377 Munich, Germany; 46 Department of Oncology and Hematology, Krankenhaus Barmherzige Brüder Regensburg, Regensburg, Germany; 47 Department of General and Visceral Surgery, Hospital Barmherzige Brüder, Regensburg, Germany; 48 Department of Medicine 1, National Center for Tumor Diseases Dresden (NCT/UCC), Sarcoma Center, University Hospital Carl Gustav Carus Dresden, Dresden University of Technology, Dresden, Germany; 49 Department of General, Visceral, Cancer and Transplant Surgery, Faculty of Medicine and University Hospital of Cologne, University of Cologne, Cologne, Germany; 50 Department of Internal Medicine II, Oncology/Hematology/BMT/Pneumology, University Medical Center Hamburg-Eppendorf, Hamburg, Germany; 51 Department of Radiation Oncology, Heidelberg University Hospital, Heidelberg, Germany; 52 Heidelberg Ion-Beam Therapy Center (HIT), Heidelberg University Hospital, Heidelberg, Germany; 53 National Center for Tumor Diseases (NCT), Heidelberg, Germany; 54 Department of Radiation Oncology, University Hospital Erlangen, Erlangen, Germany; 55 Division of Clinical Oncology, Department of Internal Medicine, Medical University of Graz, Graz, Austria; 56 Department of Hematology and Medical Oncology, University Medical Center Göttingen, Göttingen, Germany; 57 Department of Particle Therapy, University Hospital Essen, West German Proton Therapy Centre Essen (WPE), Essen, Germany; 58 Department I of Internal Medicine, Faculty of Medicine and University Hospital, University of Cologne, Cologne, Germany; 59 Faculty of Medicine and University Hospital, University of Cologne, Center for Integrated Oncology Cologne Aachen Bonn Cologne Duesseldorf (ABCD), Cologne, Germany; 60 Department of Biochemistry and Molecular Biology, University of Würzburg, Würzburg, Germany; 61 Department for Diagnostic and Interventional Radiology, University Hospital Tübingen, Tübingen, Germany; 62 Health and Medical University Potsdam, Potsdam, Germany

**Correction: Journal of Cancer Research and Clinical Oncology (2024) 151:30** 10.1007/s00432-024-06063-z

In this article, the author’s name Armin Tuchscherer was incorrectly written as Armin Tuscherer.

The original article has been corrected.

